# Process and Outcome Measures among COPD Patients with a Hospitalization Cared for by an Advance Practice Provider or Primary Care Physician

**DOI:** 10.1371/journal.pone.0148522

**Published:** 2016-02-24

**Authors:** Amitesh Agarwal, Wei Zhang, YongFang Kuo, Gulshan Sharma

**Affiliations:** 1 Division of Pulmonary, Critical Care and Sleep Medicine, Department of Internal Medicine, University of Texas Medical Branch (UTMB), Galveston, TX, United States of America; 2 Office of Biostatistics, University of Texas Medical Branch (UTMB), Galveston, TX, United States of America; 3 Sealy Center of Aging, University of Texas Medical Branch (UTMB), Galveston, TX, United States of America; Zhongshan Hospital Fudan University, CHINA

## Abstract

**Objectives:**

To examine the process and outcomes of care of COPD patients by Advanced Practice Providers (APPs) and primary care physicians.

**Methods:**

We conducted a cross sectional retrospective cohort study of Medicare beneficiaries with COPD who had at least one hospitalization in 2010. We examined the process measures of receipt of spirometry evaluation, influenza and pneumococcal vaccine, use of COPD medications, and referral to a pulmonary specialist visit. Outcome measures were emergency department (ER) visit, number of hospitalizations and 30-day readmission in 2010.

**Results:**

A total of 7,257 Medicare beneficiaries with COPD were included. Of these, 1,999 and 5,258 received primary care from APPs and primary care physicians, respectively. Patients in the APP group were more likely to be white, younger, male, residing in non-metropolitan areas and have fewer comorbidities. In terms of process of care measures, APPs were more likely to prescribe short acting bronchodilators (adjusted odds ratio [aOR] = 1.18, 95%Confidence Interval [CI] 1.05–1.32), oxygen therapy (aOR = 1.25, 95% CI 1.12–1.40) and consult a pulmonary specialist (aOR = 1.39, 95% CI 1.23–1.56), but less likely to give influenza and pneumococcal vaccinations. Patients receiving care from APPs had lower rates of ER visits for COPD (aOR = 0.84, 95%CI 0.71–0.98) and had a higher follow-up rate with pulmonary specialist within 30 days of hospitalization for COPD (aOR = 1.25, 95%CI 1.07–1.48) than those cared for by physicians.

**Conclusions:**

Compared to patients cared for by physicians, patients cared for by APPs were more likely to receive short acting bronchodilator, oxygen therapy and been referred to pulmonologist, however they had lower rates of vaccination probably due to lower age group. Patients cared for by APPs were less like to visit an ER for COPD compared to patients care for by physicians, conversely there was no differences in hospitalization or readmission for COPD between MDs and APPs.

## Introduction

The current primary care physician workforce is estimated to be inadequate to meet the needs of increasing demand[1,2]. This perceived shortage stems from the growth in the population of older adults, increased prevalence of chronic comorbidities and an additional 13 million newly-insured needing medical services under the Affordable Care Act[3]. To meet this growing need, many health systems are looking at alternative models of care by expanding the workforce of advance practice providers (APPs) [i.e., Nurse Practitioners (NPs)/Physician Assistants (PAs)] to meet the primary care needs of patients[4,5].

APPs were introduced in the US in 1967 to fill the primary care void[6]. Since then, the demand for APPs has increased. The number of PAs in the US health care system doubled between 2000 and 2010[7] and the number of NPs increased by over 75% between 2000 and 2011[8].

APPs are increasingly contributing to management of such chronic diseases as chronic obstructive pulmonary disease (COPD), diabetes, hypertension and others[9]. However, some physician organizations claim that APPs have less training and experience managing subspecialty conditions and they cannot deliver services of as high quality or as safe as those of physicians.

Findings from past studies comparing quality of care delivered by NPs versus physicians have been mixed. Two systematic reviews published in 2002 and 2005 found no appreciable differences in health outcomes between nurse-led care and physician-led care[10]^,^[11]. A meta-analysis of 24 randomized controlled trials (RCTs) of all conditions reported that NP care was associated with higher overall survival and lower rates of hospitalization, with inconclusive effects on quality of life and costs[12]. One RCT showed no significant differences in patient health status between those cared for by NPs verses physicians at 6 months in those with diabetes or asthma, and no differences in health services utilization at 6 months or 1 year[13]. Conversely, a retrospective study found that patients receiving care from PAs or NPs experienced higher rates of emergency department (ER) visits than those receiving physician care[14].

An increasing number of patients living in rural areas receive their primary care from APPs. This trend is in part due to a decreasing number of primary care physicians, the result of a growing rural-urban disparity in physician distribution[15]. In view of these changes, it is important to determine the differences in the processes, efficiency and outcomes of healthcare delivery between physicians and APPs. No national, population based study has examined the quality of COPD primary care delivered by physicians vs APPs in the US. To address this gap in knowledge, we conducted a cross sectional retrospective cohort study of Medicare COPD patients with hospitalization cared for by APPs and primary care physicians, looking for differences in the processes and outcomes of care provided under these two models of care.

## Methods

### Data source and study cohort

First, we identified all Medicare patients with COPD in 2009 and 2010 from the Centers for Medicare and Medicaid Services (CMS) Chronic Disease Data Warehouse (CCDW). Next, to select the APP cohort, we identified 100% Medicare patients who received all of their primary care from APPs in 2010 by selecting patients with billing records for two or more outpatient evaluation and management (E&M) services by an APP and with no outpatient E&M services from MDs (general practitioner, family physician, general internist or geriatrician). Next, to select the physician cohort, we identified patients cared for by primary care physicians using a 5% national sample of Medicare data. The CMS selects a random sample of 5% of Medicare beneficiaries based on the eighth and ninth digits (05, 20, 45, 70 and 95) of their health insurance claim number and this standard dataset, available for research purposes, has been shown to be representative of the whole cohort[16]. These patients had at least two outpatient billings for E&M services from MDs and no outpatient billings for E&M services from APPs in 2010. For both groups, we excluded patients aged less than 66 years, those with incomplete enrollment in Medicare Parts A, B and D in 2009 and 2010, and those whose enrollment was based on disability or end-stage renal disease. Medicare Parts A, B and D provide coverage for hospitalization, provider services and drug benefits, respectively. We also excluded patients who were covered by health maintenance organizations (HMOs) at any time in 2009 and 2010 and those who stayed in a nursing home in 2010. We limited our study cohort to those who resided in identified urban or rural areas within the nine CMS regions and who had at least one acute hospitalization in 2010. The study was approved by the University of Texas Medical Branch (UTMB) institutional review board (IRB).

### Variables

Medicare enrollment files were used to categorize subjects by age (66–74, 75–84, ≥85 years), gender (male, female), and race/ethnicity (White, Black, Hispanic/Other). A comorbidity score (0, 1, 2, ≥3) was generated using the Elixhauser comorbidity score (excluding COPD) from inpatient and outpatient billing data. Metropolitan/non-metropolitan areas were defined using Rural-Urban Continuum Codes from the US Department of Agriculture.

### Process and Outcome Measures

Our outcomes of interest included process measures such as receipt of spirometry evaluation in 2009 or 2010; receipt of influenza and pneumococcal vaccine; use of short acting beta agonist (SABA), short acting muscarinic antagonist (SAMA), long acting beta agonist (LABA), long acting muscarinic antagonist (LAMA) or inhaled corticosteroids during the given year; referral to a pulmonary specialist; and pulmonary rehabilitation. Outcome measures were number of ER visits, hospitalizations and 30-day readmissions in 2010. We also examined follow-up rates within 30 days of hospitalization from an acute care hospital.

### Statistical Analysis

Characteristics were expressed as mean ± standard deviation for continuous variables. Categorical characteristics were summarized using counts and percentages and the chi-square test was used. For each measure of processes and outcomes of care, we built a logistic regression model, adjusted for age, gender, race, Medicaid eligibility, region, metropolitan/non-metropolitan area, comorbidity score; and number of outpatient visits and hospitalizations in the previous year. Based on findings from previous studies and clinical consideration, we chose these covariates which are the potential confounding variables for studying the effect of care delivery by APPs. Analyses were performed using SAS version 9.4 (SAS Inc., Cary, NC). All reported p-values were two-sided with p<0.05 considered statistically significant.

## Results

### Patient Demographics and Cohort Characteristics

Our study cohort included 7,257 Medicare beneficiaries with COPD. Of these, 1,999 received primary care from APPs and 5,258 received primary care from MDs during the year 2010. About 77% of patients in the APP model were cared for by NPs only, 10% were cared for by PAs only and the rest received care from both types of APPs. Table 1 shows the characteristics of these COPD patients stratified by primary care provider type (APP, MD). Patients in the APP group were more likely to be Medicaid eligible (i.e., have low socioeconomic status), white, younger, male and reside in non-metropolitan areas. Higher proportions of patients with COPD cared for by MDs were from the South Atlantic region, while higher proportions of those cared for by APPs were from the South Central region. Those seen by APPs had fewer comorbidities and fewer outpatient visits in the previous year than patients seen by MDs.

**Table 1:** Baseline characteristics of COPD patients cared for by an MD or an APP during 2010.

Demographic Characteristic	Overall n(%)	MD(%)	APP(%)
**Total N**	7257	5258	1999
**Age group (years) (%)**			
66–74	36.24	32.85	45.17
75–84	45.03	46.50	41.17
85 +	18.73	20.65	13.66
**Gender (%)**Female	64.24	65.21	61.68
Male	35.76	34.79	38.32
**Race (%)**White	86.18	84.58	90.40
Black	7.48	7.97	6.20
Hispanic	3.10	3.84	1.15
Others	3.24	3.61	2.25
**Residence area (%)**^a^ Non-metropolitan	26.22	19.72	43.32
Metropolitan	73.78	80.28	56.68
**Medicaid Eligibility (%)**^b^ Yes	35.48	34.29	38.62
No	64.52	65.71	61.38
**United State regions (%)**			
New England	4.85	4.24	6.45
Middle Atlantic	11.66	13.35	7.20
East North Central	16.80	18.30	12.86
West North Central	6.89	5.33	11.01
South Atlantic	21.99	23.34	18.46
East South Central	11.26	7.95	19.96
West South Central	13.19	13.96	11.16
Mountain	3.89	3.33	5.35
Pacific	9.48	10.21	7.55
**Hospitalization in prior year (%)**			
None	46.69	46.04	48.37
1	28.06	28.39	27.16
> = 2	25.26	25.56	24.46
**Provider outpatient visits in prior year** (Mean ± Std)	14.89±10.08	15.05±9.95	14.46±10.42
(Median, Q1-Q3)	13, 8–19	13, 8–20	12, 7–19
**Number of comorbidity**^c^(Mean ± Std)	3.53±2.49	3.58±2.49	3.39±2.51
**Comorbidities(%)Yes**			
Complicated Hypertension	75.16	76.38	71.94
Uncomplicated Hypertension	15.21	16.03	13.06
Complicated Diabetes	9.54	9.57	9.45
Uncomplicated Diabetes	32.01	32.31	31.22
Neurological Disease	5.00	5.23	4.40
Hypothyroidism	18.55	18.83	17.81
Renal Failure	13.37	13.29	13.56
Liver Disease	2.23	2.47	1.60
AIDS	0.04	0.04	0.05
Metastatic Cancer	1.61	1.22	2.65
Coagulopathy	3.91	3.97	3.75
Obesity	5.62	5.48	6.00
Alcohol Abuse	1.43	1.22	2.00
Psychoses	1.38	1.52	1.00
Depression	11.27	11.11	11.71
CHF/Valve/cardiac arrhythmia	45.12	45.47	44.17

Definition of abbreviations: COPD = chronic obstructive pulmonary disease; MD = Doctor of Medicine; APP = Advance practice provider; std = standard deviation; CHF = congestive heart failure.

^**a**^**Metro/Non-Metro area:** defined by Rural-Urban Continuum Codes from the US Department of Agriculture.

^**b**^**Medicaid Eligibility:** based on whether the patient was eligible for state buy-in coverage provided by the Medicaid program for at least one month during the index year.

^**c**^**Elixhauser comorbidity**: chronic pulmonary disease, CHF, valvular disease, pulmonary circulation disorders, peripheral vascular disorders, hypertension, paralysis, other neurological disorders, diabetes-uncomplicated, diabetes-complicated, hypothyroidism, renal failure, liver disease, peptic ulcer disease excluding bleeding, AIDS (acquired immune deficiency syndrome), lymphoma, metastatic cancer, solid tumor without metastasis, rheumatoid arthritis/collagen vascular diseases, coagulopathy, obesity, weight loss, fluid and electrolyte disorders, blood loss anemia, deficiency anemia, alcohol abuse, drug abuse, psychoses, and depression.

### Process of Care Measures

Table 2 shows the process of care measures by provider type. Patients in the APP group were less likely to receive influenza vaccine (adjusted Odds Ratio [aOR] = 0.67, 95% Confidence Interval [CI] 0.60–0.75) or pneumococcal vaccination (aOR = 0.80, 95%CI 0.66–0.97) compared to patients cared for by primary care physicians. Patients cared for by APPs were more likely to be on oxygen therapy (aOR = 1.25, 95%CI 1.12–1.40) and be referred to a pulmonary specialist (aOR = 1.39, 95%CI 1.23–1.56) than those cared for by primary care physicians. Use of spirometry evaluation and pulmonary rehabilitation did not differ significantly by group. Patients in the APP group were more likely to be prescribed any short acting bronchodilator (aOR = 1.18, 95%CI 1.05–1.32). The two groups did not differ significantly in prescription of long acting bronchodilators such as LABA, LAMA or inhaled corticosteroids.

**Table 2:** Comparison of processes of care measures between MDs and APPs in patients with COPD.

	Overall (%) Yes	MD (%)	APP (%)	Adjusted p-value	Adjusted OR^a^, 95% CI (ref = MD)
**Total N**	7257	5258	1999		
Influenza vaccine (2010) ^b^	65.15	68.01	57.63	< .001	0.67 (0.60–0.75)
Pneumococcal vaccine (2010) ^b^	9.77	10.35	8.25	0.02	0.80 (0.66–0.97)
Spirometry evaluation (2009 & 2010) ^b^	48.38	48.16	48.97	0.49	1.04 (0.93–1.16)
Oxygen therapy (2010) ^c^	43.25	41.21	48.62	< .001	1.25 (1.12–1.40)
Pulmonary specialist visit (2010)	38.86	37.30	51.38	< .001	1.39 (1.23–1.56)
Pulmonary rehabilitation (2010)	0.30	0.27	0.40	0.35	1.58 (0.61–4.11)
Long acting beta agonist prescription (LABA)	6.31	5.93	7.30	0.10	1.20 (0.97–1.49)
Long acting muscarinic antagonist prescription (LAMA)	30.23	30.09	30.62	0.77	0.98 (0.87–1.11)
Short acting beta agonist prescription(SABA)	47.42	46.50	49.82	0.22	1.07 (0.96–1.20)
Short acting muscarinic antagonist prescription(SAMA)	9.85	9.51	10.76	0.48	1.07 (0.89–1.28)
LABA and inhaled corticosteroid prescription	39.58	39.46	39.87	0.62	1.03 (0.92–1.15)
Inhaled corticosteroid prescription	13.83	13.62	14.41	0.65	1.04 (0.89–1.21)
Any long acting bronchodilator	59.39	58.96	60.53	0.30	1.06 (0.95–1.19)
Any short acting bronchodilator	63.54	62.02	67.53	0.01	1.18 (1.05–1.32)
No medication	22.98	23.62	21.31	0.20	0.92 (0.80–1.05)

Definition of abbreviations: COPD = chronic obstructive pulmonary disease; MD = Doctor of Medicine; APP = Advance practice provider; CI = Confidence interval; CHF = Congestive Heart Failure; OR = Odds ratio.

**Adjusted OR**^**a**^: Logistic regression model were used to estimate odds ratio, adjusted by age, gender, race, region, metro/non-metro area, Medicaid Eligibility, elixhauser comorbidity score, outpatient visit in the previous year and hospitalization in the previous year.

Influenza vaccine^**b**^, Pneumococcal vaccine^**b**^, Spirometry evaluation^**b**^: were identified from physician professional file and outpatient facility file.

Oxygen therapy^**c**^: was identified from Durable Medical Equipment (DME) file.

### Outcome Measures

Table 3 shows the outcome measures for patients receiving primary care from MDs or APPs. Patients receiving care from APPs had lower rates of ER visits for COPD (aOR = 0.84, 95%CI 0.71–0.98), lower follow-up rate with primary care physician (aOR = 0.38, 95% CI 0.33–0.43) and high follow up rate with pulmonary specialist within 30 days of hospitalization for COPD (aOR = 1.25, 95%CI 1.07–1.48) than those cared for by an MD. Patients receiving APP care had a slightly lower odds of 30-day readmission (aOR = 0.96, 95% CI 0.83–1.11), slightly lower odds of any ER visits (aOR = 0.95, 95%CI 0.83–1.08) than those receiving care from an MD, but the difference was not statistically significant. The two groups did not differ in total number of acute care hospitalizations in 2010 (1.75±1.25 vs 1.69±1.15, p value 0.09) in the MD and APP group, respectively.

**Table 3:** Outcomes of COPD patients cared for by a primary care physician or an Advance Practice Provider (APP) during 2010.

	Overall (Yes)	MD	APP	Adjusted p Value	Adjusted OR^a^, 95% CI (ref = MD)
**Total N**	7257	5258	1999		
ER visit in 2010 (%)	79.37	80.07	77.54	0.43	0.95 (0.83–1.08)
ER visit for primary COPD in 2010 (%)	14.21	14.59	13.21	0.03	0.84 (0.71–0.98)
30-day readmission in 2010 (%)	17.24	17.25	17.21	0.59	0.96 (0.83–1.11)
Pulmonary specialist visit within 30 days after COPD hospitalization (%)^b^	18.58	17.89	20.36	0.01	1.25 (1.07–1.48)
Primary care physician visit within 30 days after COPD hospitalization (%)^b^	67.22	73.03	52.09	< .001	0.38 (0.33–0.43)
Primary care physician or Pulmonary visit within 30 days after COPD hospitalization (%)^b^	73.51	77.97	61.89	< .001	0.44 (0.39–0.51)

Definition of abbreviations: COPD = chronic obstructive pulmonary disease; MD = Doctor of Medicine; APP = Advance practice provider; CI = Confidence interval; OR = Odds ratio; ER = emergency department.

**Adjusted OR**^**a**^: Logistic regression model were used to estimate odds ratio, adjusted by age, gender, race, region, metro/non-metro area, Medicaid Eligibility, elixhauser comorbidity score, outpatient visit in the previous year and hospitalization in the previous year.

^b^This population was patients who had COPD hospitalization by 11/30/2010.

## Discussion

In a sample of Medicare beneficiaries with COPD with a hospitalization in 2010, we found that APPs were more likely to prescribe short acting bronchodilators or oxygen therapy and to consult a pulmonary specialist, but less likely to give influenza and pneumococcal vaccinations compared to MDs. Patients receiving care from APPs had lower rates of ER visits for COPD and a higher follow-up rate with a pulmonologist within 30 days of hospitalization for COPD than those cared for by an MD.

NP/PAs were introduced in the US in the 1960s; since then, demand for NP/PAs has exceeded the supply. Approximately 205,000 NPs and >93,000 PAs practice in the US[7,17]. About half are employed in primary care settings (defined as family medicine, general medicine and general pediatrics)[18]. It is estimated that APPs could provide care for 50–90% of patients presenting to primary care[19]. With the increasing number of APPs as primary care providers, they will be more likely to be called upon to manage patients with such chronic conditions as COPD. Large regional differences across US in patients with COPD cared for by APPs are likely representative of state regulations on NP practices[20,21].

The present study showed no differences in hospitalization or readmission of COPD patients by group. This result is consistent with previous studies of chronic disease management by NPs/PAs. An RCT study showed no difference in blood pressure or total cholesterol control between patients receiving care from NPs and those receiving care from primary care physicians[21]. Similarly, studies of diabetic patients showed no difference in HbA_1_C control and outcomes in patients cared for by NPs or primary care physicians[22]. A recent study showed similar outcomes for in-hospital mortality for patients receiving care from APPs and physicians in intensive care units[23].

Our study showed more use of resources such as pulmonary referrals, oxygen therapy and medication prescription in the APP group, consistent with findings from diabetic care studies, which showed higher use of referrals and resources in patients cared for by APPs than in those cared for by MDs[24]. Similarly, a recent study showed greater use of imaging services by APPs compared to MDs[25]. The more frequent specialist consults with NP care may be due to the recognized need for expertise and skills outside of the NP’s scope of practice for complex patients. Lower use of influenza vaccination in the APP group is likely related to the lower age group of these COPD patients under their care.

Contrary to our hypothesis that patients cared for by APPs have better access to care, we found lower rates of follow-up clinic visits after acute hospitalization in the APP group than in the primary care physician group. However, patients cared for by APPs had more clinic follow-up visits with a pulmonary specialist than the patients of MDs. Higher follow-up rates with pulmonologist post hospitalization in APP group may partly explain the lower trends in emergency visits and readmission. Studies have shown that early follow up with a pulmonary physician is associated with lower readmission rates[26,27]. Previous studies including a Cochrane meta-analysis have shown that patients receiving care from APPs have a higher frequency of return visits compared to patients of physicians[28,29] Higher follow-up rates with a primary care provider in the physician group were likely due to the greater accessibility of physicians compared to APPs. We excluded patients with COPD who received care under a mixed NP/MD model. The lower follow-up rates for APP patients can be explained by the higher number of patients under NP/PA care who may follow up with a physician after hospitalization, thus resulting in lower follow-up rates in the APP group.

Our study showed no difference in the 30-day readmission rate after acute COPD hospital admission in patients cared for by APPs vs primary care physicians. No intervention has yet been proven to reduce readmissions in COPD patients. A recent systemic review found inadequate evidence to recommend specific interventions to reduce readmissions in this population[30]. Jennings et al., in a recent randomized controlled trial, showed no difference in 30-day risk of rehospitalization or ED visits after implementation of COPD bundle at discharge. The elements of the bundle were smoking cessation counseling; screening for gastroesophageal reflux disease, depression and anxiety; standardized inhaler teaching; and a 48-hour post-discharge phone call[31].

This study has several limitations. First, we were not able to distinguish whether APPs were working independently or under physician supervision, as our definition of NP care was based on E&M billing. However, we included only patients for whom all bills for outpatient visits in a given year originated from either APPs only or MDs only. Second, assessing processes and outcomes of care in an observational study is subject to selection bias; for example, severity of COPD was not measured, a factor that can affect outcomes of care. Due to the cross sectional nature of the study, the use of spirometry and vaccinations (specifically, pneumococcal vaccination) were lower than in prior reports[32] We examined only pneumococcal vaccination rates during the study period and missed the opportunity to capture the true rates, given the infrequent recommendations compared to influenza vaccination. Third, we did not look at outcomes and processes of care for COPD patients cared for by both APPs and primary care physicians. Future research should examine the benefits of shared model in managing patients with COPD compare to APPs vs MDs model alone. Complex patients are more likely to benefit from shared model of care than either solo model. Shared models provide easy access to care and expertise needed to manage these patients.

Fourth, we did not account for cost of care in the two different care models. Previous studies have shown that APP cost of care is the same as or slightly lower than that of a physician[33]. Fifth, we did not compare patient satisfaction for the two groups as in previous RCTs comparing APP vs physician models; however, this is a limitation of the observational study design. Sixth, the results are not generalizable to patients younger than 65 years and those who do not have complete enrollment in Medicare Part A, B and D. Seventh, we reported the adjusted effect estimates but cannot exclude the possibility of false positive findings given the multiple testing. Finally, the proportion of patient with COPD cared for by APPs in the current study is higher than in the general population, as we used the 100% Medicare population with COPD cared for by APPs.

In summary, compared to patients cared for by primary care physicians, patients cared for by APPs were more likely receive short acting bronchodilators, oxygen therapy and being referred to a pulmonologist. Despite lower rates of influenza and pneumococcal vaccination among patients with COPD cared for by APPs, these patients were less like to visit an ER for COPD compared to those cared for by primary care physicians.
